# Exploring Variations in Physical and Chemical Characteristics of *Barringtonia* Nuts: A Novel Forest Food

**DOI:** 10.3390/foods14122147

**Published:** 2025-06-19

**Authors:** Shahla Hosseini Bai, Bruce Randall, Repson Gama, Basil Gua, Doni Keli, Peter Brooks, Brittany Elliott, Helen M. Wallace

**Affiliations:** 1 School of Environment and Science, Griffith University, Nathan, Brisbane, QLD 4111, Australia; 2 School of Science, Technology and Engineering, University of the Sunshine Coast, Maroochydore, QLD 4558, Australia; 3 Beg’s Sol Seed Technology and Supplies, Honiara, Solomon Islands; 4 Solfarm Fresh, Honiara, Solomon Islands; solfarm.fresh@gmail.com; 5 School of Biology and Environmental Science, Faculty of Science, Queensland University of Technology, Brisbane, QLD 4000, Australia

**Keywords:** *Barringtonia*, domestication, commercialisation, tree selection, post-harvest, kernel to fruit percentage

## Abstract

Food security remains one of the most critical global challenges of the 21st century. Traditional tree crops domesticated by indigenous people have the potential to increase food security and improve the livelihoods of smallholders in developing countries. However, the nut characteristics of many traditional tree crop species in the tropics are poorly understood. In particular, physical and chemical characteristics are important to explore when selecting trees to commercialise. Three species, *Barringtonia procera*, *B. edulis*, and *B. nova-hiberniae*, have a long history of traditional use and domestication in Pacific Island countries. The aim of this study was to explore the physical and chemical characteristics of *Barringtonia* spp. in three Pacific countries: Solomon Islands, Vanuatu, and Fiji. There were significant differences in kernel weight, oil concentration, and fatty acid concentration among the countries. The kernel weight was significantly higher in Solomon Islands compared with those in Vanuatu and Fiji (9.65 g, 7.61 g, and 5.64 g, respectively). Average kernel weight in Fiji was well above 3 g, which indicated that processing could be commercially viable. The total oil concentration was significantly higher in Vanuatu and Solomon Islands than Fiji, with average concentrations of 38.96% in Solomon Islands, 47.11% in Vanuatu, and 26.20% in Fiji. *Barringtonia* spp. exhibited high concentrations of unsaturated fatty acids, similar to other tropical nuts, which suggests that it may be a potential healthy oil for human consumption. Notably, kernel size, oil concentration, and fatty acid composition varied geographically, potentially due to climatic differences and historical seed transfer. Our study demonstrated the potential of *Barringtonia* to be commercialised to enhance food and nutrition security and provide a guide for cultivar selection.

## 1. Introduction

Food security for a growing global population remains one of the key challenges of the current century [1]. Underutilised food sources, such as forest foods used by indigenous cultures, provide one option to increase food security, particularly in developing countries [2,3,4,5,6]. Tree nuts, in particular, are a forest food that can improve food security and nutrition, since they are high in protein, essential nutrients, and beneficial fatty acids compared to other food sources [7,8,9]. The daily consumption of tree nuts has been recommended as part of a healthy diet to reduce heart disease and decrease cholesterol [10,11]. In addition, tree nuts are high in micronutrients and therefore can help to address hidden hunger such as iron and folate deficiencies. They can be dried, processed, and commercially sold to distant markets where perishable food crops are not feasible. Indigenous trees nuts have been domesticated in many traditional cultures over thousands of years [2,7], but few of these have also been widely commercialised, especially in the tropics [5]. Some examples of tropical tree nuts that have been commercialised include *Canarium indicum*, *C. ovatum*, *Macadamia integrifolia*, and *Bertholletia excelsa* [4,7,12,13].

The successful commercialisation of tree nut species requires trees with large kernels and a high kernel-to-fruit ratio; otherwise, commercialisation will not be economically viable [14,15]. A large kernel mass and/or a high kernel-to-fruit ratio increase financial returns for processors, growers, and smallholder farmers [4,16,17,18]. Tree nuts exhibit high within-species variability in their kernel attributes, for example, in kernel size, kernel recovery, and nutritional composition [16,18,19,20,21]. Additionally, the kernel attributes of a species can further be affected by environmental conditions. Therefore, it is important to explore the variability of kernel attributes collected from a broad geographic range when a tree species is targeted for commercialisation.

Tree nuts are high in oil and are rich in essential fatty acids [10,21,22,23]. Usually, tropical tree nuts are rich in unsaturated fatty acids [24]. Traditionally, tree selection and breeding programmes are based on many factors such as kernel size, resistance to environmental variables, and flowering time [16,18,25,26]. Recently, kernel chemical composition has attracted attention as a possible factor for tree selection and breeding [18,27]. Kernel chemical composition can drive both the health benefits and shelf life of the kernel [7,28,29]. Usually, tropical tree nuts are rich in unsaturated fatty acids that confer health benefits [8,30]. The main dominant unsaturated fatty acids in tropical tree nuts are linoleic acid (C18:2) and linolenic acid (C18:3) [24,30,31]. However, both oil content and fatty acid profile can vary within cultivars, species, growing conditions, and origins [27]. The fatty acid composition of many underutilised indigenous tree nuts remains unknown.

*Barringtonia* spp. are tree nuts with a long history of traditional use in Pacific Island countries. There are three species of *Barringtonia* with kernels (seeds) that are considered edible and are a staple food source for communities in the Pacific: *B. procera* (Miers) Knuth, *B. edulis* Seem, and *B. nova-hiberniae* Laut [32,33,34]. These three species have been intensively selected and hybridised for desirable fruit and tree characteristics in indigenous food systems, and many different morphotypes are now cultivated [32]. *B. procera* occurs in East New Guinea, Solomon Islands, and Vanuatu, while *B. edulis* occurs in Fiji, Vanuatu, and Solomon Islands and *B. nova-hiberniae* occurs in Papua New Guinea, Solomon Islands, and Vanuatu [32,35]. The large kernel size makes this plant highly desirable for food security and nut commercialisation. However, there may be large differences in kernel attributes throughout the geographical species distribution due to indigenous selection [16,18].

In this study, we examined the fruit and kernel physical and chemical characteristics of *Barringtonia* spp. in Solomon Islands, Vanuatu, and Fiji. These three countries represent the key regions within the distribution range of the three *Barringtonia* species of economic importance in the south-west Pacific [36,37] and capture the genetic diversity and environmental variability of Barringtonia spp. In particular, we aimed to (a) explore the kernel mass, fruit mass, and kernel-to-fruit ratio (%) of *Barringtonia* spp. between the three Pacific countries, (b) understand the relationship between kernel size and fruit size, and (c) compare total oil concentrations and fatty acid concentration attributes between the three Pacific countries. Our results can be used to inform the selection and management of *Barringtonia* spp. for nutrition security and commercialisation.

## 2. Materials and Methods

### 2.1. Sample Collection and Preparation

**Figure 1:**
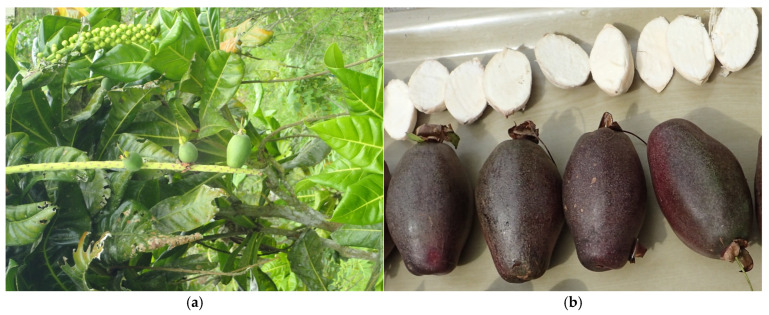
(**a**) Unripe fruit hanging from tree and (**b**) ripe fruit and kernel of *Barringtonia* spp. (Photo credit: Bruce Randall).

The fruit of *Barringtonia* spp. (Figure 1) were randomly collected from the canopy of trees at various locations in the Solomon Islands, Vanuatu, and Fiji. In brief, we had 330 replicated fruit samples collected from 66 trees within three countries. Fruit samples of *Barringtonia* spp. were collected from each tree with five replicates. In total, 160 samples from Solomon Islands (32 trees × 5 replicates per tree), 70 samples from Vanuatu (14 trees × 5 replicates per tree), and 100 samples from Fiji (20 trees × 5 replicates per tree) were collected in this study. The fruit samples were collected over 24 months between March 2017 and 2019.

The three *Barringtonia* spp. examined in this study are predominantly found in coastal areas and secondary tropical rainforests at low elevations, typically up to 600 m above sea level, across the southwest Pacific region [37,38]. The fruiting season of these species varies across geographic regions in the Pacific [37]. In lower-latitude countries such as Solomon Islands, fruiting can occur irregularly with up to two or three fruiting events per year [37]. In contrast, fruit production in Fiji and Vanuatu tends to be more seasonally consistent, and is typically limited to one or two fruiting events annually [37]. As a result, fruit collection for this study occurred at varying times, depending on local fruiting patterns.

Each fresh fruit was individually weighed using digital scales (PA4101 Pioneer Analytical Balance, OHAUS, Parsippany, NJ, USA), and then the kernels were extracted manually from the fruit and also individually weighed. Each fruit contained only one kernel. The kernels were dried using a dehydrator or an oven at approximately 40 °C to reach a moisture concentration of 6%. Samples were then sent to Australia for further analysis. We also calculated the kernel-to-fruit percentage as given in Equation (1):

Kernel-to-fruit ratio (%) = (Kernel weight/fruit weight) × 100(1)

### 2.2. Oil Extraction and Chemical Analyses

The kernel samples (*n* = 25 per tree) were randomly divided into five replicates for analysis, with each replicate containing five kernels per tree. Oil was extracted from kernels of each replicate as follows: approximately 1.5–2.5 g of kernels was ground using a mortar and pestle/hand-held garlic press and transferred to a pre-weighed beaker. The weight of each beaker and nut sample was recorded. A volume of 45–50 mL Pentane was used for the first oil extraction while stirring for 30–45 min using a magnetic stirrer. The mixture was transferred to 50 mL Falcon tube, and centrifuged at 2600 rpm for 6 min at 4 °C. The supernatant was transferred to pre-weighed round-bottomed flask. A second oil extraction was performed by adding 35–40 mL fresh Pentane to the pellet, and the mixture was stirred and centrifuged as described above, and supernatant was transferred to the flask. After evaporating the pentane for 12 min in Bucci Rotovac (BÜCHI Labortechnik AG, Switzerland), the extracted oil in the round bottomed flasks was weighed and then transferred into glass vials and stored at 4 °C for FAMES analysis. Oil concentration was determined by expressing the weight of extracted oil as a percentage of the weight of nuts used in extraction as given in Equation (2).

Percentage oil = (mass of oil × 100)/mass of kernel(2)

Fatty acid methyl esters (FAMEs) were prepared using acid catalysis and analysed using gas chromatography–mass spectrometry (GC-MS) according to the method described by Hamilton et al. [39] with minor modifications. The GC-MS instrument was a PerkinElmer Clarus 580 GC coupled to a SQ 8S MS (PerkinElmer, Waltham, MA, USA). The column used was an Elite-5MS (30 m × 0.25 mm × 0.25 μm). The helium carrier gas had a constant flow of 1.0 mL/min. The injection port was 300 °C with split ratio of 100:1 for −0.5 to 2.0 min, then 30:1 thereafter [7]. The temperature program was operated from 50 °C for 0.5 min, ramping at 10 °C/min until 300 °C and holding for 1.0 min [7]. The mass spectrometer analysed a mass range from 40 to 400 (*m*/*z*) from 3.1 to 26.5 min at 70 eV [7]. The NIST spectral database was used for matching retention sequences. Integration software was used to measure peak areas which were then expressed as a percentage of total detected FAME peaks. The sum of myristic acid (C14:0), palmitic acid (C16:0), stearic acid (C18:0), arachidic acid (C20:0), and C22:0 (behenic acid) constituted the total saturated fatty acids. The sum of palmitoleic acid (C16:1 cis), linoleic acid (C18:2), oleic acid (C18:1 cis), elaidic acid (C18:1 trans), and eicosenoic acid (C20:1) constituted the total unsaturated fatty acids (TUSFAs).

### 2.3. Statistical Analysis

We calculated the average fruit mass, kernel mass, and kernel-to-fruit ratio from individual fruit data for each tree. We assessed differences in fruit mass, kernel mass, kernel-to-fruit ratio, and oil content among samples collected from the three countries. Due to unequal sample sizes and the evidence of heterogeneity of variances (Levene’s test, *p* < 0.05), Welch’s one-way ANOVA was employed to test for significant differences in group means [40]. Following a significant Welch’s ANOVA result (*p* < 0.05), Games–Howell post hoc tests were performed to identify pairwise differences between countries. This approach was chosen because it does not assume equal variances or equal sample sizes, making it appropriate for the structure of our dataset [41]. A principal component analysis (PCA) was implemented to visualise how fatty acid compositions including C16:1, C18:2, C18:1 cis, C20:1, C14:0, C16:0, C18:0, C20:0, and C22:0 were distributed based on the countries. All data analyses were completed using SPSS version 24 (IBM Corp. Chicago, IL, USA).

## 3. Results

**Figure 2:**
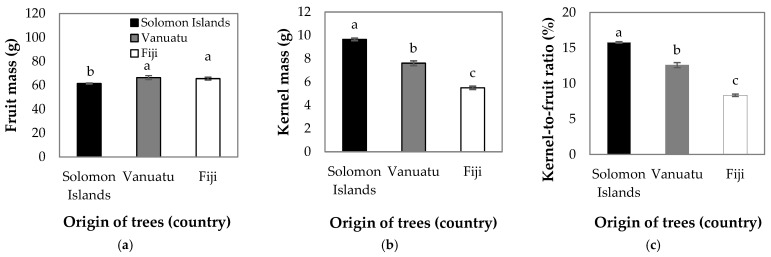
(**a**) Fruit mass, (**b**) kernel mass, and (**c**) kernel-to-fruit ratio in *Barringtonia* spp. collected from Solomon Islands (black columns), Vanuatu (grey columns), and Fiji (white columns). Mean and standard errors are presented (*n* = 160 in Solomon Islands; *n* = 70 in Vanuatu; *n* = 100 in Fiji). Different lower-case letters indicate differences among countries at *p* < 0.05.

**Figure 3:**
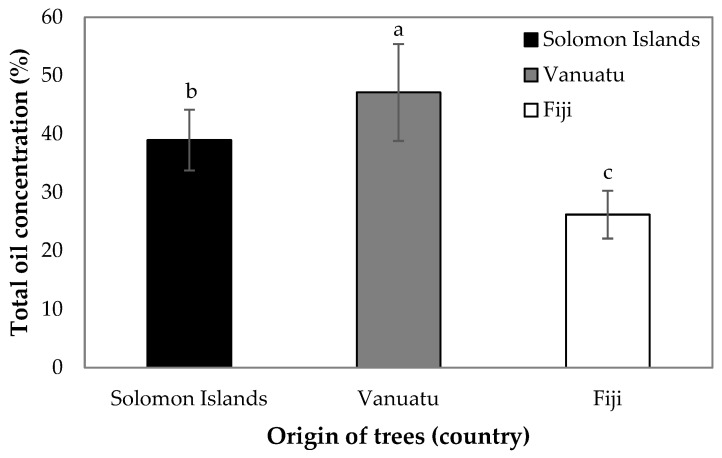
Total oil concentration in *Barringtonia* spp. nuts collected from Solomon Islands (black symbols), Vanuatu (grey symbols), and Fiji (white symbols). Mean and standard errors are presented (*n* = 160 in Solomon Islands; *n* = 70 in Vanuatu; *n* = 100 in Fiji). Different lower-case letters indicate differences among countries at *p* < 0.05.

We found that Vanuatu and Fiji had significantly larger *Barringtonia* spp. fruit than Solomon Islands (Figure 2a). In contrast, *Barringtonia* spp. from Solomon Islands had significantly larger kernels and these kernels were almost double the size of those from Fiji (Figure 2b). Consequently, the kernel-to-fruit ratio in Solomon Islands was also the greatest and was on average around 4% larger compared with Vanuatu (Figure 2c). A significant positive relationship was observed between fruit weight and kernel weight (Figure 3). The fruit weight explained 41%, 13%, and 46% of the variations observed in kernel weight collected from Solomon Islands, Vanuatu, and Fiji, respectively (Figure 3).

The total oil concentration extracted from kernels was significantly higher in Vanuatu compared with Solomon Islands and Fiji, and the total oil of kernels collected from Fiji was significantly lower than both Vanuatu and Solomon Islands (Figure 3). Individual trees in Solomon Islands had significant differences in total oil extracted (Supplementary Table S1). The total oil concentration in Solomon Islands was on average 38.96%, ranging between 27.91% and 53.78% (Figure 3). The total oil in Vanuatu varied significantly between trees, ranging from 31.95% to 68.18%, with an average of 47.11% (Supplementary Tables S1 and S3). The total oil of individual trees in Fiji did not significantly differ and averaged 26.20%, ranging from 20.24% to 36.67% (Supplementary Tables S1 and S3).

**Figure 4:**
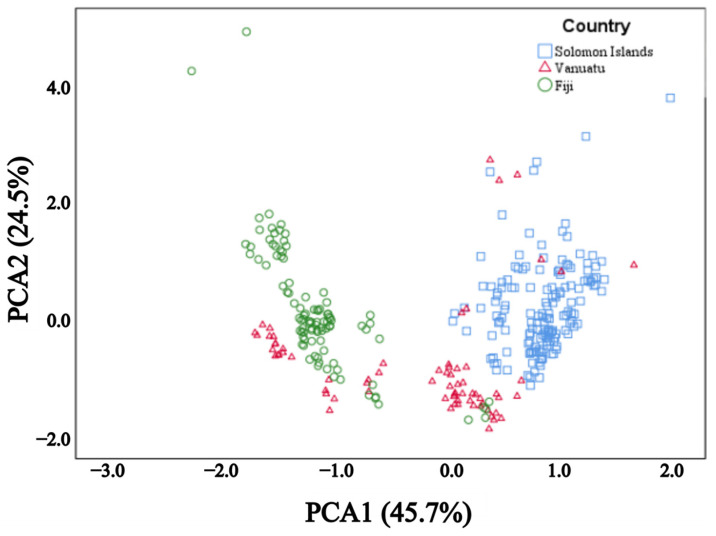
Principal component analyses (PCA) showing the clustering of *Barringtonia* spp. based on their fatty acid composition per country of origin: Solomon Islands (open blue rectangular), Vanuatu (open red triangular), and Fiji (open green circle).

In general, the total unsaturated fatty acids were found in higher concentrations than total saturated fatty acids (Table 1). Five forms of saturated fatty acids and five forms of unsaturated fatty acids were detected (Table 1). The dominant saturated fatty acid was C16:0 (palmitic acid), followed by C18:0 (stearic acid), C20:0 (arachidic acid), C14:0 (myristic acid), and C22:0 (behenic acid) (Table 1). C18:1C (oleic acid) was the dominant unsaturated fatty acid and C18:2 (linoleic acid) was the second most dominant unsaturated fatty acid in Solomon Islands and Vanuatu, whereas C18:2 had greater concentrations than C18:1C in Fiji (Table 1). The total saturated fatty acid concentrations were significantly higher in Solomon Islands compared with the other two countries (Table 1). In contrast, the total unsaturated fatty acid concentrations were significantly lower in Solomon Islands than the other two countries (Table 1). The total saturated and unsaturated fatty acid concentrations varied significantly among individual trees in all three countries (Supplementary Table S2). The fatty acid composition between Solomon Islands and Fiji had distinct clusters (Figure 4).

## 4. Discussion

Our results showed that the kernel size of *Barringtonia* spp. in all countries met the threshold for commercialisation. Notably, kernel weight was significantly higher in Solomon Islands compared with those in Vanuatu and Fiji. Since larger kernels and higher kernel-to-fruit ratios enhance profitability for both farmers and processors, this trait is particularly valuable for commercialisation efforts [4,14,42,43]. A kernel mass of over 3 g for the newly commercialised *C. indicum* has been suggested to be economically viable [18]. The average kernel mass of *Barringtonia* spp. was well above 3 g even in Fiji. The smaller kernel size observed in Fiji may be attributed to the presence of *Barringtonia edulis*, a species endemic to the region. Several environmental factors are also known to influence interpopulation variability in plants [44]. Generally, this can include the origin of the nuts, local climate and environmental conditions, pollination dynamics, soil properties, nutrient availability, and genetics [16,19,20,45,46]. Significant variability between trees within each country for kernel mass have been reported in other Pacific nut-producing species such as *Terminalia catappa* [16] and *C. indicum* [18]. Variation in kernel properties is common in tree nuts and is often reported between trees of different genotypes, and even within a single genotype [16,18]. In our complimentary study, we also found high tree-to-tree variations in the kernel weight which might be driven by local domestication [16]. Hence, this study clearly highlights that there is potential to select and propagate trees with large kernels to increase the commercial returns of *Barringtonia* spp. in all countries [18,47].

Total unsaturated fatty acid concentrations were higher than saturated fatty acid concentrations. The fatty acid composition of the *Barringtonia* spp. in this study was very similar to other tropical nuts including *T. catappa* [48] and *C. indicum*, an indigenous timber tree in the Pacific that has been recently commercialised [4,12,49,50]. The high unsaturated fatty acid concentrations make these nuts a healthy choice for consumption [10,28]. Consuming foods that are high in unsaturated fatty acids has been associated with decreased cholesterol and lower rates of heart diseases [9,28,51]. Our PCA indicated that the fatty acid profile of our kernel samples was segregated based on the country of origin suggesting the fatty acid profiles are affected by the origin of the samples. For example, interestingly, the abundance of unsaturated fatty acids was higher in nuts collected from Vanuatu and Fiji than in those from Solomon Islands. Foods with high concentrations of unsaturated fatty acids are counted as healthy choices and play an important role to maintain cholesterol and minimise heart diseases [10].

We found differences between countries of up to approximately 5 g for kernel size, 21% for oil concentration, and 15% of total unsaturated fatty acid concentration. Variation in fruit size and quality is common, has been reported even at tree levels, and is driven by different factors [16,19,20,45,46,49]. All three countries experience distinct tropical climatic conditions. As a result, interannual climate variability and inconsistencies in seasonal timing during sample collection may have partly contributed to the observed variability in kernel size and fatty acid composition among countries. The distinct clustering of fatty acid compositions between Solomon Islands and Fiji could also be partly attributed to variations in the cultivars of *Barringtonia* spp. [27]. In Pacific countries, it is common for villagers to have intensively selected and propagated trees with desirable traits, particularly those with food or economic benefits like *Barringtonia* spp. for domestication [6,36,52]. There are many different morphotypes of *Barringtonia* sp. that have been cultivated in traditional agroforestry systems in Pacific countries [32]. Over generations, seeds may have been exchanged between islands and hence contributed to a diversity of *Barringtonia* spp. cultivars [32].

## 5. Conclusions

Our findings suggest that *Barringtonia* spp. kernels contain oils rich in beneficial fatty acids, supporting their potential as a nutritious food source [7]. Our study highlights the significance of identifying local selections for propagation to enhance commercial returns and improve food and nutrition security in rural communities. Notably, *Barringtonia* spp. trees from Solomon Islands produced the largest kernels compared to other countries. However, trees from Vanuatu and Fiji also yielded kernel sizes that are considered commercially viable. This study can help guide the commercialisation of *Barringtonia* spp., aiming to empower smallholders and contributing to improved nutrition and food security.

## Figures and Tables

**Table 1:** Individual fatty acid concentrations, total saturated fatty acid concentrations (TSFA), and total unsaturated fatty acid concentrations (TUSFA) of oils in *Barringtonia* spp. nuts collected from Solomon Islands, Vanuatu, and Fiji. Mean and standard errors are presented (*n* = 160 in Solomon Islands; *n* = 70 in Vanuatu; *n* = 100 in Fiji). nd: not detected. Different lower-case letters indicate differences among countries for that variable at *p* < 0.05.

	Solomon Islands	Vanuatu	Fiji
C14_0	0.11 ± 0.0034 a	0.043 ± 0.0042 b	0.03 ± 0.0011 c
C16_0	39.46 ± 0.2349 a	29.40 ± 0.8748 b	21.80 ± 0.4054 c
C18_0	6.06 ± 0.0573 c	8.84 ± 0.3819 b	9.47 ± 0.1693 a
C20_0	0.41 ± 0.0082 a	0.26 ± 0.0180 b	0.30 ± 0.0081 b
C22_0	0.05 ± 0.0019 a	0.04 ± 0.0024 a	0.04 ± 0.0040 a
C16_1	0.12 ± 0.0046 a	0.03 ± 0.0036 b	0.02 ± 0.0011 c
C18_1C	32.58 ± 0.2274 b	37.77 ± 0.4744 a	32.13 ± 0.7948 b
C18_1T	0.86 ± 0.0231 a	0.29 ± 0.0326 b	nd
C20_1	0.05 ± 0.0019 a	0.03 ± 0.0025 b	0.05 ± 0.0037 a
C18_2	20.33 ± 0.2484 c	23.32 ± 0.6568 b	36.16 ± 0.6598 a
TSFA	46.08 ± 0.2339 a	38.57 ± 0.5467 b	31.64 ± 0.4199 c
TUSFA	53.94 ± 0.2358 c	61.44 ± 0.5478 b	68.36 ± 0.4199 c

## Data Availability

The original contributions presented in this study are included in the article/Supplementary Materials. Further inquiries can be directed to the corresponding author.

## Supplementary Materials

The following supporting information can be downloaded at: https://www.mdpi.com/article/10.3390/foods14122147/s1, Table S1: Mean and standard errors of total oil percentage in *Barringtonia* spp. in Solomon Islands, Vanuatu, and Fiji. Table S2: Mean and standard errors of total saturated fatty acid (TSFA) and total unsaturated fatty acid (TUSFA) concentrations in *Barringtonia* spp. Solomon Islands, Vanuatu and Fiji. Table S3: Descriptive statistics of *Barringtonia* spp. kernel attributes in each country. Means, minimum and maximum values are presented. Table S4: Descriptive statistics of total saturated fatty acid (TSFA) and total unsaturated fatty acid (TUSFA) concentrations in *Barringtonia* spp. kernels collected from the Solomon Islands, Vanuatu and Fiji.
