# Supplementary material for: Exploring Variations in Physical and Chemical Characteristics of Barringtonia Nuts: A Novel Forest Food
Source: Foods. 2025 Jun 19;14(12):2147. doi: 10.3390/foods14122147 (PMC12192473; doi:10.3390/foods14122147)
Supplement: Supplementary file 1 [file foods-14-02147-s001.zip › foods-3643352-supplementary.pdf]

Supplementary Table S1: Mean and standard errors of total oil percentage in *Barringtonia* spp. in Solomon Islands, Vanuatu, and Fiji. Differences among trees in their total oil were based on one-way ANOVA, $p<0.05$ in each country. Modified least significant differences of Solomon Islands:1.37, Vanuatu: 0.92, Fiji: 0.29.

Tree Number	Solomon Islands	Vanuatu	Fiji
1	32.38 ± 1.10 no	32.97 ± 0.43 k	24.60 ± 0.19 a
2	30.02 ± 0.85 o	41.51 ± 1.69 hi	26.32 ± 0.17 a
3	33.79 ± 1.06 mn	49.76 ± 0.17 d	28.13 ± 0.21 a
4	35.36 ± 1.10 klm	45.19 ± 0.19 f	24.68 ± 0.14 a
5	34.38 ± 0.75 lmn	42.05 ± 0.14 gh	28.00 ± 0.22 a
6	31.88 ± 0.62 no	43.42 ± 0.15 g	24.01 ± 0.12 a
9	40.16 ± 1.65 efg	40.25 ± 0.49 ij	36.23 ± 0.17 a
10	35.91 ± 0.35 jklm	54.61 ± 0.20 b	27.71 ± 0.17 a
11	39.88 ± 0.47 efg	53.79 ± 0.25 bc	28.36 ± 0.16 a
12	37.43 ± 0.97 hijk	39.66 ± 0.20 j	29.83 ± 0.12 a
13	35.72 ± 0.40 klm	67.39 ± 0.21 a	28.07 ± 0.17 a
14	36.63 ± 0.64 ijkl	52.17 ± 0.39 c	21.16 ± 0.29 a
15	40.41 ± 0.59 efg	46.94 ± 0.31 e	23.33 ± 0.15 a
16	43.30 ± 0.32 cd	48.62 ± 0.67 d	34.94 ± 0.13 a
17	40.41 ± 1.17 efg		20.97 ± 0.14 a
18	41.93 ± 0.71 cde		24.78 ± 0.07 a
19	43.37 ± 0.99 cd		23.94 ± 0.07 a
20	38.66 ± 0.62 ghi		25.96 ± 0.19 a
21	40.27 ± 0.32 efg		22.19 ± 0.29 a
22	38.40 ± 0.35 ghij		20.84 ± 0.17 a
23	38.43 ± 0.44 ghij		
24	41.99 ± 1.32 cde		
25	41.42 ± 1.01 def		
26	49.79 ± 0.46 a		
27	48.90 ± 1.64 ab		
28	47.09 ± 0.38 b		
29	32.60 ± 0.66 n		
30	32.48 ± 0.50 no		
31	44.22 ± 0.58 c		
32	46.75 ± 0.81 b		
33	38.80 ± 0.78 fghi		
34	37.95 ± 0.28 ghijk		

Supplementary Table S2: Mean and standard errors of total saturated fatty acid (TSFA) and total unsaturated fatty acid (TUSFA) concentrations in *Barringtonia* spp. Solomon Islands, Vanuatu and Fiji. Differences among trees in their TSFA and TUSFA were significant at each country based on one-way ANOVA, $p<0.05$. Modified least significant differences of TSFA: Solomon Islands:0.53, Vanuatu :0.55, Fiji: 0.39; modified least significant differences of TUSFA: Solomon Islands:0.54, Vanuatu:0.55, Fiji:0.39.

	Solomon Islands						Vanuatu						Fiji					
Tree No	TSFA			TUSFA			TSFA			TUSFA			TSFA			TUSFA		
1	40.91	0.48	o	59.09	0.48	a	44.14	0.27	a	55.86	0.27	i	32.38	0.16		67.63	0.16	fg
2	41.34	0.15	o	58.66	0.15	ab	42.73	0.88	b	57.27	0.88	h	31.91	0.13		68.09	0.13	ef
3	44.58	0.45	ijk	55.42	0.45	fgh	35.75	0.18	f	64.45	0.16	d	29.97	0.11		70.03	0.11	d
4	43.79	0.81	kl	56.21	0.81	def	41.23	0.25	c	58.77	0.25	g	32.10	0.20		67.90	0.20	ef
5	41.65	0.27	no	58.35	0.27	ab	39.68	0.10	d	60.32	0.10	f	33.02	0.11		66.98	0.11	g
6	44.28	0.43	jkl	55.72	0.43	efg	32.58	0.19	h	67.42	0.19	b	31.41	0.08		68.59	0.08	e
7	45.07	0.52	hij	54.93	0.52	ghi	41.51	0.16	c	58.49	0.16	g	24.66	0.39		75.34	0.39	b
8	47.33	0.46	de	52.67	0.46	lm	34.50	0.34	g	65.50	0.34	c	23.85	0.35		76.15	0.35	a
9	45.36	0.18	hi	54.64	0.18	hi	32.65	0.08	h	67.35	0.08	b	27.89	0.25		72.11	0.25	c
10	51.09	0.26	a	48.91	0.26	p	43.91	0.60	a	56.09	0.60	i	29.87	0.51		70.13	0.51	d
11	46.55	0.09	efg	53.45	0.09	jkl	30.39	0.15	i	69.61	0.15	a	38.35	0.37		61.65	0.37	i
12	49.85	0.12	bc	50.15	0.12	no	43.59	0.16	ab	56.41	0.16	hi	30.29	0.24		69.71	0.24	d
13	49.26	0.19	c	50.74	0.19	n	39.40	0.12	d	60.60	0.12	f	24.65	0.23		75.35	0.23	b
14	49.80	0.20	bc	50.20	0.20	no	37.96	0.04	e	62.04	0.04	e	36.33	0.17		63.67	0.17	h
15	47.80	0.68	d	52.20	0.68	m							38.56	0.16		61.44	0.16	i
16	48.89	0.27	c	51.11	0.27	n							38.27	0.19		61.73	0.19	i
17	47.22	0.21	de	52.78	0.21	lm							31.91	0.15		68.09	0.15	ef
18	45.78	0.19	gh	54.22	0.19	ij							32.96	0.21		67.04	0.21	g
19	46.92	0.25	def	53.08	0.25	klm							32.48	0.15		67.52	0.15	fg
20	51.63	0.12	a	48.37	0.12	p							31.87	0.12		68.13	0.12	ef
21	45.94	0.26	fgh	54.06	0.26	ijk												
22	43.62	0.13	kl	56.38	0.13	def												
23	46.58	0.21	efg	53.42	0.21	jkl												
24	49.19	0.15	c	50.81	0.15	n												
25	46.74	0.24	efg	53.26	0.24	jkl												
26	50.67	0.28	ab	49.33	0.28	op												
27	43.58	0.27	kl	56.88	0.31	cd												
28	45.78	0.28	gh	54.22	0.28	ij												
29	42.52	0.24	mn	57.71	0.37	bc												
30	43.37	0.23	lm	56.63	0.23	de												
31	44.03	0.31	kl	55.97	0.31	def												
32	43.48	0.25	lm	56.52	0.25	de												

Supplementary Table S3: Descriptive statistics of *Barringtonia* spp. kernel attributes in each country. Means, minimum and maximum values are presented.

		Fruit weight (g)	Kernel weight (g)	Kernel recovery (%)	Total oil (%)
**Solomon Islands**	Mean	61.41	9.65	15.79	38.96
	N	860	860	860	163
	Minimum	30.39	0	0	27.91
	Maximum	128.62	20.99	29.03	53.78
**Vanuatu**	Mean	66.38	7.61	12.59	47.11
	N	254	254	254	67
	Minimum	30.24	1.25	2.49	31.95
	Maximum	171.68	17.27	29.47	68.18
**Fiji**	Mean	65.52	5.49	8.34	26.2
	N	358	358	358	100
	Minimum	13.8	0	0	20.24
	Maximum	177.64	14.81	25.94	36.67

Supplementary Table S4: Descriptive statistics of total saturated fatty acid (TSFA) and total unsaturated fatty acid (TUSFA) concentrations in *Barringtonia* spp. kernels collected from the Solomon Islands, Vanuatu and Fiji.

		C14_0	C16_0	C18_0	C20_0	C22_0	C16_1	C18_2	C18_1C	C18_1T	C20_1	TSFA	TUSFA
**Solomon Islands**	Mean	0.11	39.46	6.06	0.41	0.05	0.12	20.33	32.58	0.86	0.05	46.08	53.94
	N	160	160	160	160	160	160	160	160	160	160	160	160
	Minimum	0.03	32.66	4.82	0.25	0.01	0.04	13.46	26.72	0.27	0.02	39.66	48.15
	Maximum	0.22	45.62	8.07	0.85	0.14	0.62	28.68	39.84	1.56	0.17	51.85	60.34
	Variance	0.002	8.828	0.526	0.011	0.001	0.003	9.873	8.274	0.085	0.001	8.756	8.899
	Std. Error<br>of Mean	0.0034275<br>5	0.2348890<br>3	0.0573316<br>7	0.0082081<br>2	0.0019033<br>5	0.0045785<br>5	0.2484119<br>8	0.2274005	0.0230665<br>2	0.0018510<br>5	0.23393	0.23583
	Range	0.188528	12.96847	3.253045	0.607803	0.136111	0.579324	15.220392	13.121294	1.291218	0.154614	12.19	12.19
**Vanuatu**	Mean	0.043	29.395499<br>9	8.8384217<br>8	0.2603371<br>5	0.0354704<br>6	0.0337360<br>2	23.315253<br>8	37.773037<br>5	0.2854939<br>3	0.0339657<br>1	38.5728	61.4415
	N	70	70	70	70	70	70	70	70	70	70	70	70
	Minimum	0.006817	17.011004	0.632059	0.13626	0.007394	0.005936	15.350152	27.190518	0	0.0079	30.17	54.37
	Maximum	0.177733	38.3855	15.116291	0.822728	0.106227	0.160145	32.989618	45.677332	0.955063	0.108013	45.63	69.83
	Variance	0.001	53.573	10.207	0.023	0	0.001	30.202	15.755	0.074	0	20.923	21.005
	Std. Error<br>of Mean	0.0042069<br>4	0.8748336<br>5	0.3818514	0.0179639<br>3	0.0024351<br>6	0.0036217<br>5	0.6568499<br>1	0.4744196<br>6	0.0326124<br>7	0.0024757<br>8	0.54672	0.54779
	Range	0.170916	21.374496	14.484232	0.686469	0.098833	0.154209	17.639466	18.486814	0.955063	0.100113	15.46	15.46
**Fiji**	Mean	0.030	21.801003<br>6	9.4676078<br>8	0.2952138<br>8	0.0427033<br>8	0.0185614<br>8	36.164596<br>4	32.126367<br>5	0	0.0541680<br>4	31.6363	68.3637
	N	100	100	100	100	100	100	100	100	100	100	100	100
	Minimum	0.014044	14.400872	4.680286	0.105804	0.017103	0.003425	21.578483	17.050287	0	0.024953	22.72	60.91
	Maximum	0.067039	31.712377	12.224296	0.46512	0.420276	0.057675	47.224739	46.720586	0	0.399412	39.09	77.28
	Variance	0	16.437	2.867	0.007	0.002	0	43.532	63.164	0	0.001	17.631	17.631
	Std. Error<br>of Mean	0.0010720<br>9	0.4054238<br>8	0.1693321<br>1	0.0080924	0.0039969<br>9	0.0010871<br>6	0.6597881<br>3	0.7947583<br>2	0	0.0037455<br>5	0.4199	0.4199
	Range	0.052995	17.311506	7.544011	0.359315	0.403173	0.054251	25.646256	29.670299	0	0.374459	16.37	16.37
